# Co-Operative Production

**Published:** 1898-05-14

**Authors:** 


### Modern Sociology.

### CO-OPERATIVE PRODUCTION.

"The spirit of despair with regard to the possibilities of co-operative production which resulted from the numerous failures of some years ago is giving way before the growing success of the movement to-day." With these words the report of the Labour Association, which met at Kettering, begins its report. It goes on to say that while, when the association began its work, there were only 15 businesses working strictly on the co-operative principle in the country, there are now upwards of 200, and that the growth in capital, trade, and profits have been even greater than the increase in the number of businesses. To those of us who believe that co-operation forms the golden mean between monopoly on the one hand and socialism on the other, such news as this is very satisfactory; but still, one cannot but feel that a total of even 200 businesses is not much for productive co-operation to show, considering the time the principle has been in vogue, and the energy that has been devoted to developing it. One cannot but ask why, while co-operative distribution has been a striking success, co-operative production, the influence of which on economic problems is far more important, should still lag far behind.

Co-operative distributive stores have for the most part succeeded best in those businesses into which personal taste enters least, in the purveying of groceries and such goods, where, on the whole, everyone wants the same article. Even where they have not entirely eliminated the competition of private traders, they have caused the latter to reduce their prices very considerably; so much so, indeed, that the small trader, who had few sales and took large profits, has been almost entirely destroyed, and the only real rival of the co-operative store is the large merchant, or joint-stock company, which, with a large capital, can push its business widely, command many sales, and fully satisfy its shareholders with a dividend of 5 or 6 per cent. But co-operative stores have not yet eliminated those warehouses which cater in any degree to personal taste. There still remain many shops where people are willing to pay an excessive price, if the mere utility of the article purchased be considered, because it is unique or exceptionally tasteful.

But co-operative factories are few, and small in comparison with the businesses owned and controlled by private owners. What is the reason of this? We think that most discussions of this undeniable fact are rather confused by the division of the factors of production into only two classes—labour and capital. These two combine, indeed, but only through the medium of a third—the employer or *entrepreneur*, who utilises the accumulations of capital, and pays the wages of labour. Most economists talk of the employer as the capitalist, but in the majority of cases he owns only a part of the capital employed in running his business. The remainder he borrows, sometimes from private individuals, sometimes from a bank—in which case some of his own workmen may be among his creditors—but in every case it is the same. He carries on his business not because he is a capitalist—whether he owns much or little of the capital employed in his business—but as a master, an adventurer, as we have called him, an *entrepreneur*. What the *entrepreneur* gains by his labour of is, first, what are called the wages of management or supervision, plus a variable remainder which we call profit. This last it is that represents his reward for undertaking the risks of a business and making himself responsible for the payment of wages and of interest on borrowed capital, and guaranteeing the safety of the money borrowed. He has great responsibilities, great anxieties; these stimulate him to great exertions, and he expects a great recompense. This recompense we call profit, and it varies immensely according to the capacity of the individual *entrepreneur*. You cannot keep such a man, as a rule, as mere manager of another's business; he wants independence, to try his own methods, to push his own way, and stand or fall by his own ideas. These are the men who, when their capacity is equal to their ambition, make those great fortunes which arouse the envy of their workers, and lead them to desire to enter the field of co-operative production.

Now, if a number of workmen desirous of forming a co-operative business in their own line of work can obtain the services of such a man, and pay him well enough to make him content to be their servant instead of his own master, they are fortunate. But will they do so? The average workman knows the value of his own work, but has little conception of the importance of any labour that does not involve the soiling of the hand. Will a man whose wages amount only to £1 a week, and his share of profit to a few shillings more, be content to give the manager of his business £1,000 a year? Will he not grudge the manager's salary just as formerly he grudged the master's profit, and bring it down to a point which will make the manager throw up his situation? He will get another manager, it is true, but the latter may be one without the capacity of his predecessor, a man who, as a private manufacturer, would come to bankruptcy, or at best earn only a small and precarious living, and who will not be able to conduct the co-operative factory with such an amount of success as will satisfy its owners, who, just because the money they have invested in it is their little all, will be more exacting in the matter of dividends than an ordinary capitalist. In short, is it not likely that the working man will at last argue with himself thus: "If I work for a private employer, I get my wage just the same as I do now. My master can dismiss me, it is true; but he is not likely to do so if I do my work well, and, if I do not, my mates, who are joint managers of this business with me, will do the same. If I can save anything off my wages, I am free to invest it in any way I choose, and I have a claim against anyone to whom I lend it for the amount, and the interest he has agreed to pay me for the use of it. I get interest on my money when I invest it in the co-operative factory, but the interest is variable and uncertain in amount, and if the business does not succeed my money will be lost, and I have no claim against anyone for repayment. It is better for me, since I cannot have sole control of the business, in which case my share of the profit might be large enough to make it worth while for me to take the risk, to keep my work separate from my investment, and not to combine with either the responsibilities of the *entrepreneur*." Such reasoning is not often uttered; but that it is acted upon is proved by the fact that even in Rochdale, where there are mills owned by working men, these prefer to work in one factory and own shares in another. They prefer to keep the functions of labour and capital distinct. Nevertheless, in small businesses which cater for a local market, and where no exceptional capacity in management is required, co-operative production may succeed well enough; but we see no signs of its competing with those great businesses which find their markets as often on the other side of the globe as at home, and demand for their success a business capacity that is akin to genius.

